# Supplementary material for: CARM1-expressing ovarian cancer depends on the histone methyltransferase EZH2 activity
Source: Nat Commun. 2018 Feb 12;9:631. doi: 10.1038/s41467-018-03031-3 (PMC5809368; doi:10.1038/s41467-018-03031-3)
Supplement: Supplementary file 1 — Supplementary Information [file 41467_2018_3031_MOESM1_ESM.pdf]

Supplementary Figure 1

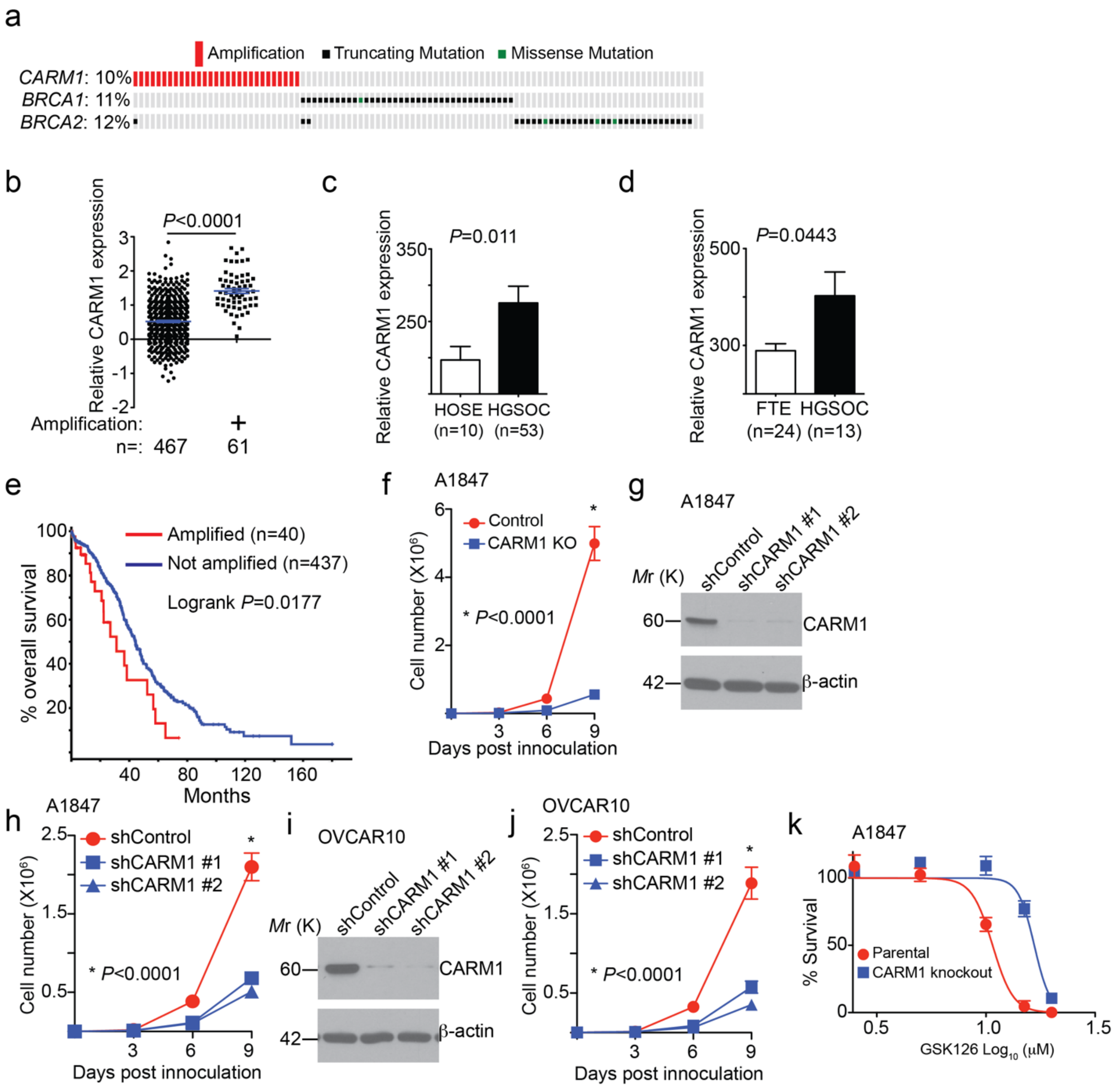

Supplementary Figure 1. *CARM1* is amplified in high-grade serous ovarian carcinomas (HGSOC) and CARM1 inhibition suppresses the growth of EOC cells.

(a) *CARM1* amplification and *BRCA1/2* mutation profiles in the published TCGA HGSOC database [1].

(b) Relative levels of CARM1 expression in the TCGA HGSOC cases with or without CARM1 amplification. *P*-values are from two-tailed *t*-test.

(c) Relative expression of *CARM1* in laser capture and microdissected (LCM) high-grade serous ovarian cancer (HGSOC) and normal human ovarian surface epithelial (HOSE) cells in a published dataset [2]. *P*-values are from two-tailed *t*-test.

(d) Relative expression of *CARM1* in laser captured and microdissected HGSOC and fallopian tube epithelial (FTE) cells in a published dataset [3]. *P*-values are from two-tailed *t*-test.

(e) Overall survival of EOC patients with or without *CARM1* amplification based on TCGA copy number analysis of HGSOC cases with survival data (n=477). *P*-value was calculated by log-rank test.

(f) Growth curves of parental control and CARM1 knockout A1847 cells. Mean of three independent experiments with SEM. *P*-values are from two-tailed *t*-test.

(g) Expression of CARM1 in A1847 cells expressing the indicated shCARM1 or controls.

(**h**) Growth curves of control and CARM1 knockdown A1847 cells. Mean of three independent experiments with SD. *P*-values are from two-tailed *t*-test.

(i-j) Same as g-h, but for CARM1 high OVCAR10 EOC cells. Mean of three independent experiments with SD. *P*-values are from two-tailed *t*-test.

(k) GSK126 dose-response curves for parental and CARM1 knockout A1847 cells determined by colony formation assay. Mean of three independent experiments with SEM. *P*-values are from two-tailed *t*-test.

Supplementary Figure 2

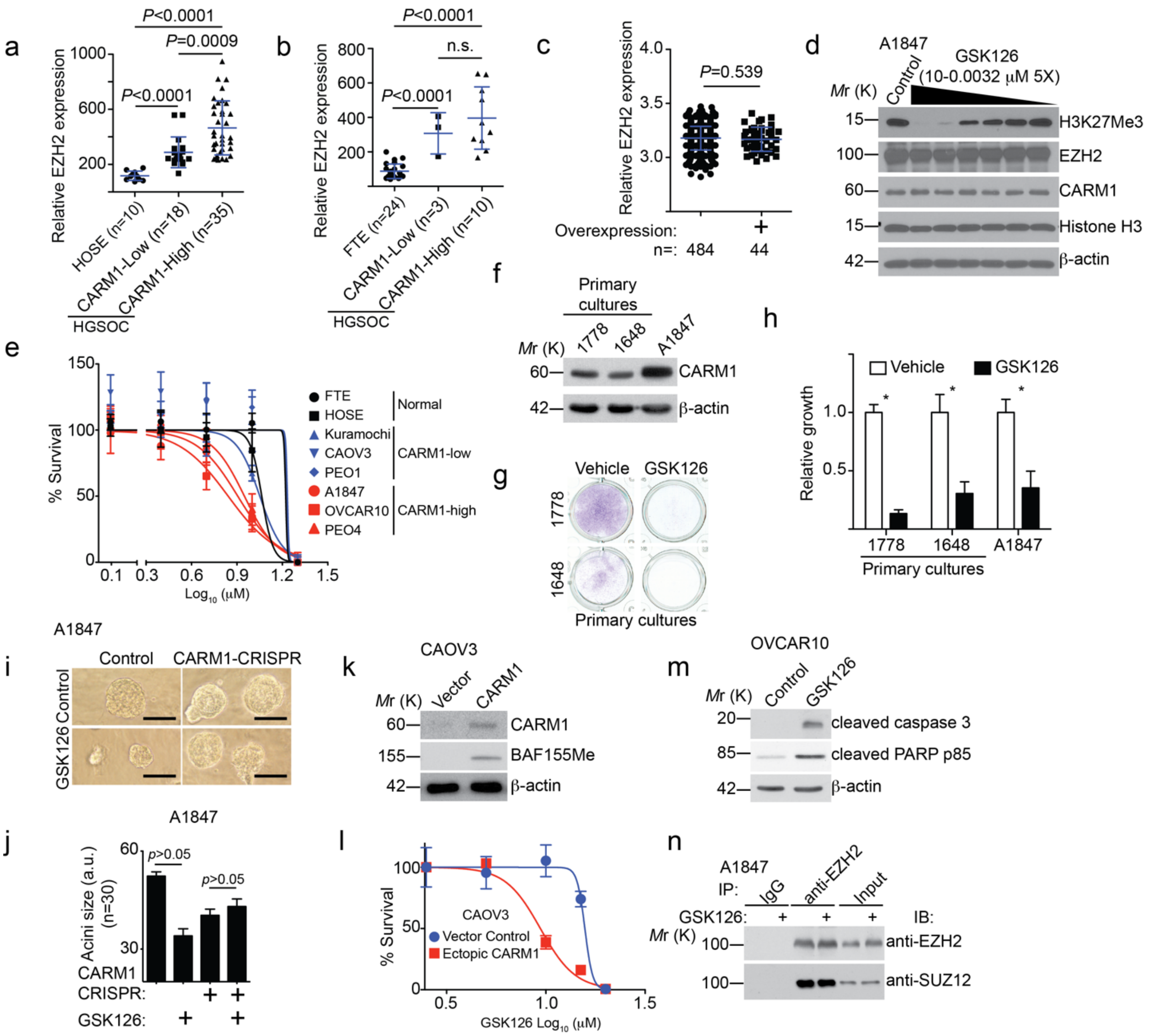

Supplementary Figure 2. CARM1-expressing EOC cells are selectively sensitive to EZH2 inhibition.

(a) Relative expression of *EZH2* in human ovarian surface epithelial (HOSE, n=10) cells and laser capture-microdissected high-grade serous ovarian carcinomas (LCM HGSOC) with high *CARM1* (n=35) or low *CARM1* (n=18) expression in a published dataset [2]. Error bars represent SD. *P* value was calculated by two-tailed t test.

(b) Relative expression of *EZH2* in fallopian tube epithelial cells (n=24) and LCM HGSOC with high *CARM1* (n=10) or low *CARM1* (n=3) expression in a published dataset [3]. Error bars represent SD. *P* value was calculated by two-tailed t test.

(c) Relative *EZH2* expression in the TCGA HGSOC cases with (n=44) or without (n=484) *CARM1* overexpression. Note that *CARM1* amplified cases were analyzed in Fig. 1f and are excluded from this analysis. Relative *EZH2* expression levels were transformed to $\log_2$ (10+expression) values. *CARM1* overexpression in the TCGA HGSOC dataset was defined as *CARM1* expression with Z-score>2 and without concurrent *CARM1* amplification. Error bars represent SD. *P* value was calculated by two-tailed t test.

(d) EZH2 inhibitor GSK126 decreases H3K27Me3 levels in a dose-dependent manner. A1847 cells were treated with the indicated concentrations of GSK126 for 72 hours and examined for expression of the indicated proteins by immunoblot. Expression of histone H3 and β-actin was used as a loading control.

(e) GSK126 dose-response curves for the indicated normal, CARM1-low and CARM1-high EOC cells as determined by colony formation assay. Mean of three independent experiments with SEM.

(f) Expression of CARM1 in primary cultures of HGSOCs was determined by immunoblot. Expression of b-actin was used as a loading control. A1847 cell line was used as a positive control for CARM1 expression.

(g) Equal number of cells from the indicated primary cultures of HGSOCs were treated with 10μM GSK126 or vehicle control for 7 days and stained for colony formation using 0.05% crystal violet.

(h) Quantification of **(g)**. Error bars represent SD and N=4. * *P*<0.001. A1847 cell line was used as a positive control

(i) Representative images of acini formed by indicated cells treated with or without 10 μM GSK126 in 3D cultures using Matrigel extracellular matrix for 12 days. Scale Bars = 50 of measurable units (AU) using the NIH Image J software.

(j) Quantification of the diameter of acini formed by the indicated cells with or without 10 μM GSK126 treatment in 3D culture for 12 days. Error bars represent SEM and n=30 acini per sample.

(k-l) Expression of CARM1, BAF155Me and β-actin in CAOV3 cells with or without ectopic CARM1 expression (k). GSK126 dose-response curves of the indicated cells were determined by colony formation (l). Mean of three independent experiments with SEM.

(m) CARM1-high OVCAR10 cells were treated with 10 μM GSK126 or vehicle DMSO control for 7 days. The expression of apoptosis markers cleaved caspase 3 and cleaved PARP p85 was determined by immunoblot. Expression of β-actin was used as a loading control.

(**n**) EZH2 inhibitor GSK126 did not affect PRC2 subunits EZH2 and SUZ12 interaction in CARM1-high A1847 cells. A1847 cells were treated with 10 μM GSK126 or vehicle DMSO control for 7 days. The cells were subjected to immunoprecipitation (IP) analysis by using an anti-EZH2 antibody. The IP'd product was examined for EZH2 and SUZ12 expression by immunoblot. An isotype-matched IgG was used as a control.

Supplementary Figure 3

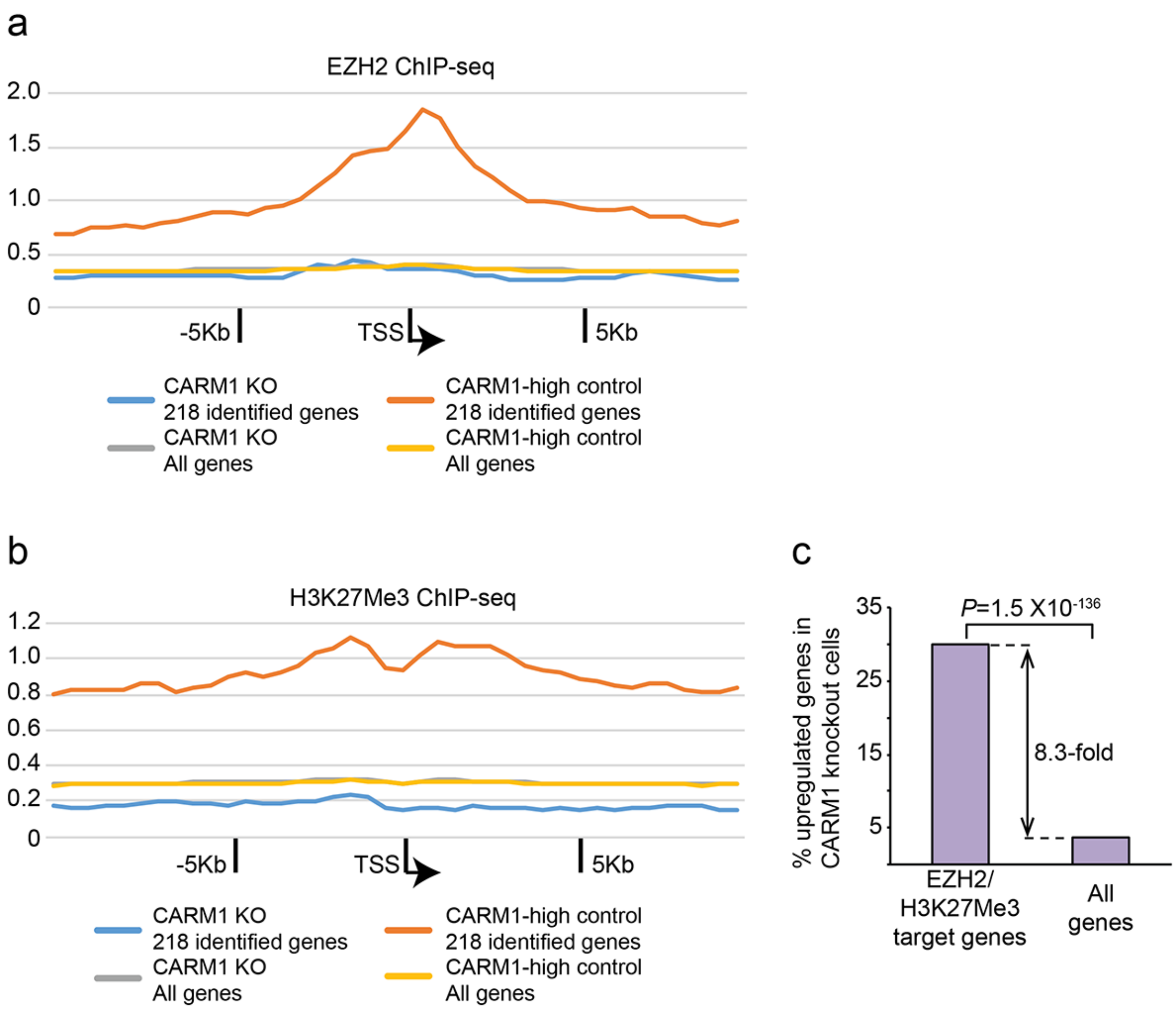

Supplementary Figure 3. EZH2/H3K27Me3 target genes are enriched in genes upregulated by CARM1 knockout in A1847 cells.

(a-b) ChIP-seq peaks compilation of EZH2 (a) and H3K27Me3 (b) relative to transcription starting site (TSS) of the 218 direct EZH2/H3K27Me3 target genes that are upregulated in CARM1 knockout cells compared with control CARM1-high A1847 cells. **(c)** EZH2/H3K27Me3 direct target genes are enriched in genes upregulated by CARM1 knockout.

Supplementary Figure 4

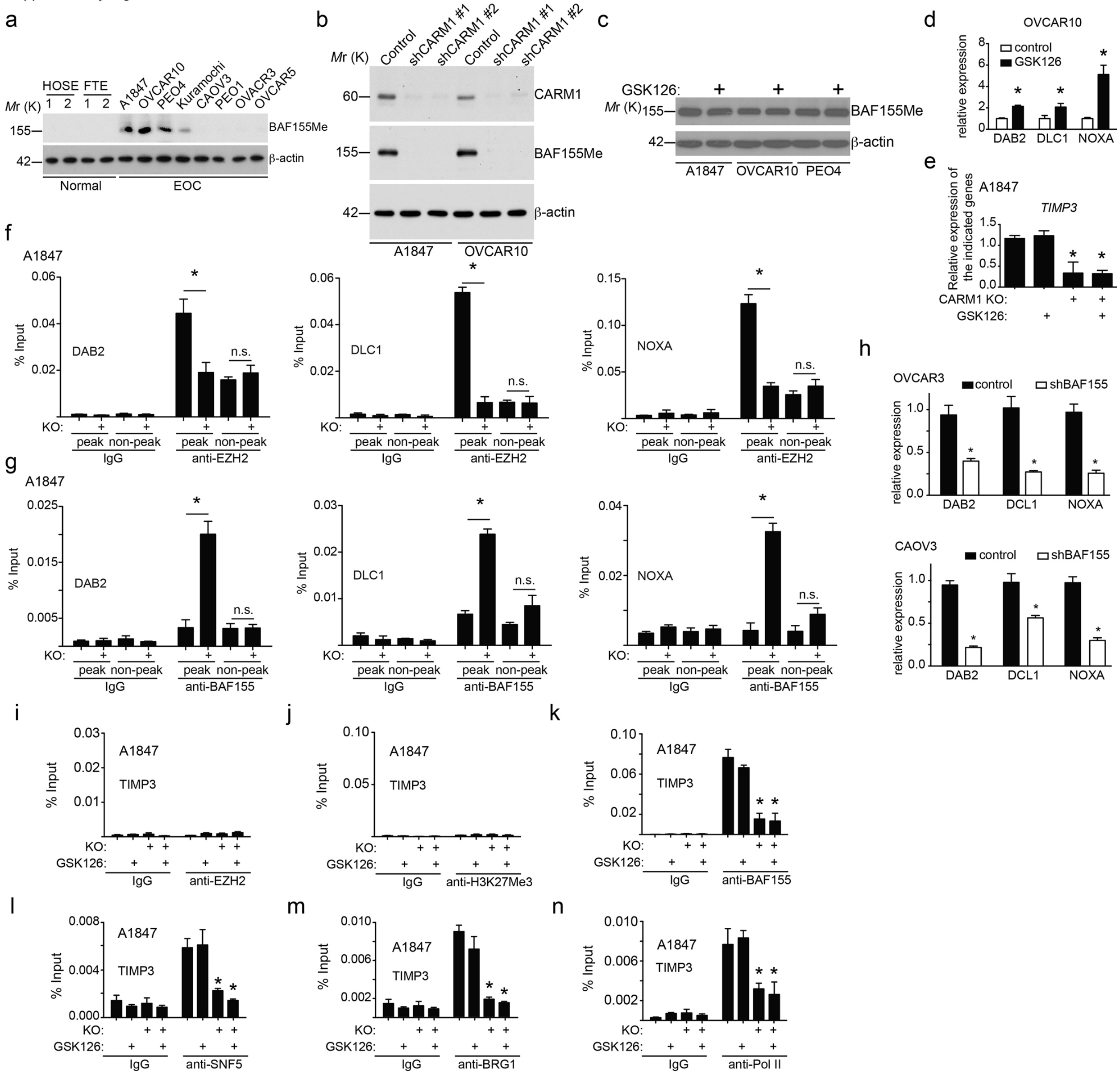

Supplementary Figure 4. CARM1 regulates BAF155Me and EZH2 inhibition promotes the expression of BAF155/EZH2 target genes but not BAF155Me target genes.

(a) Expression of BAF155Me in the indicated EOC cell lines, HOSE and FTE cells was determined by immunoblot. Expression of β-actin was used as a loading control.

(b) The indicated CARM1-high cells were infected with the indicated shCARM1 or controls. Expression of CARM1, BAF155Me levels and a loading control (β-actin) was determined by immunoblot.

(c) The indicated cells were treated with 10 μM GSK126 or vehicle control for 7 days. Expression of BAF155 and a loading control β-actin was determined by immunoblot.

(d) CARM1-high OVCAR10 cells treated with 10 μM GSK126 or vehicle DMSO control for 7 days. Expression of the indicated BAF155/EZH2 target genes was determined by qRT-PCR. * $P<0.001$. Mean of three independent experiments with SEM. *P*-values are from two-tailed *t*-test.

(e) Parental control and CARM1 knockout A1847 cells were treated with 10 μM GSK126 or vehicle controls. mRNA expression of BAF155Me target gene TIMP3 was determined by qRT-PCR. * $P<0.001$. Mean of three independent experiments with SEM. *P*-values are from two-tailed *t*-test.

(f-g) Parental control and CARM1 knockout A1847 cells were treated with 10 μM GSK126 or vehicle DMSO controls. The cells were subjected to ChIP analysis using anti-EZH2 (f) or anti-BAF155 (g) antibodies. An isotype-matched IgG was used as a negative control. ChIP products were subjected to qPCR analysis using primers specific for the EZH2 ChIP-seq peak promoter regions of the indicated genes. qPCR using primers for a region with non-EZH2 ChIP-seq peak (2Kb upstream of the transcription starting site) region was used as a negative control. Mean of 3 independent experiments with SEM. * $P<0.04$ compared with controls. n.s.: not significant. *P*-values are from two-tailed *t*-test.

(h) The indicated CARM1-low EOC cells with or without shBAF155 expression were examined for expression of the indicated genes. * $P<0.001$ compared with controls. *P*-values are from two-tailed *t*-test.

(i-n) Parental control and CARM1 knockout A1847 cells were treated with 10 μM GSK126 or vehicle controls. The cells were subjected to chromatin immunoprecipitation (ChIP) analysis using anti-EZH2 (i), anti-H3K27Me3 (j), anti-BAF155 (k), anti-SNF (l), anti-BRG1 (m) or anti-RNA Pol II (n) antibodies. An isotype-matched IgG was used as a negative control. ChIP products were subjected to qPCR analysis using primers specific for the promoter regions of the human

TIMP3 genes. Mean of 3 independent experiments with SEM. * *P*<0.001 compared with controls. *P*-values are from two-tailed *t*-test.

Supplementary Figure 5

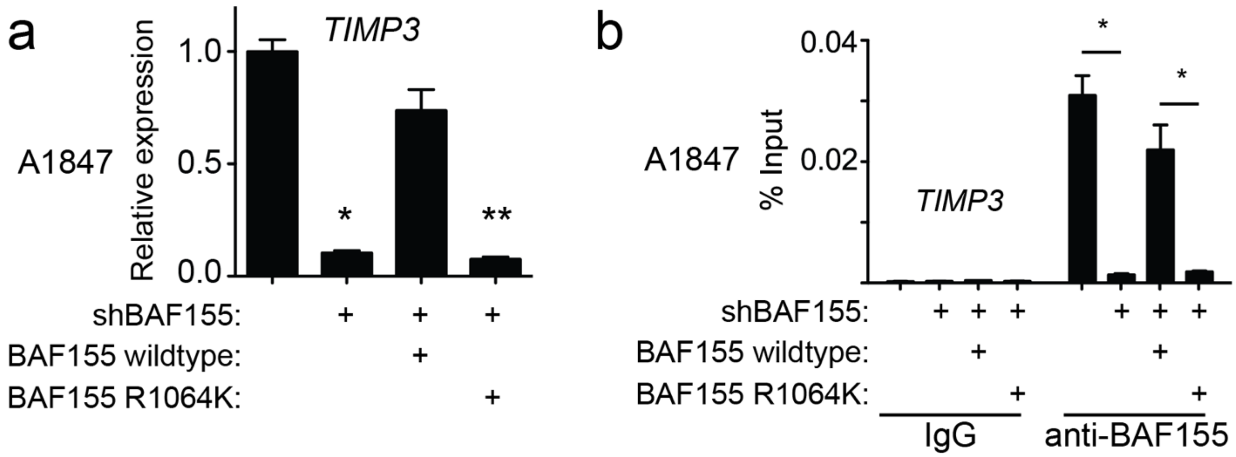

Supplementary Figure 5. CARM1 promotes the expression of BAF155Me target genes.

(a) CARM1-expressing A1847 cells were infected with a lentivirus encoding shBAF155 targeting the 3' untranslated region (UTR) of the human *BAF155* gene together with a retrovirus encoding wild-type BAF155 (WT) or a BAF155 R1064K mutant. Expression of BAF155Me target gene *TIMP3* was determined by qRT-PCR in the indicated cells. Mean of three independent experiments with SEM. * *P*<0.001. *P*-values are from two-tailed *t*-test.

(b) Same as a, but the cells were subjected to ChIP analysis using an anti-BAF155 antibody. An isotype matched IgG was used as a negative control. ChIP products were subjected to qPCR analysis using primers specific for the promoter of the human *TIMP3* gene. Error bars represent SEM. * *P*<0.001. *P*-values are from two-tailed *t*-test.

Supplementary Figure 6

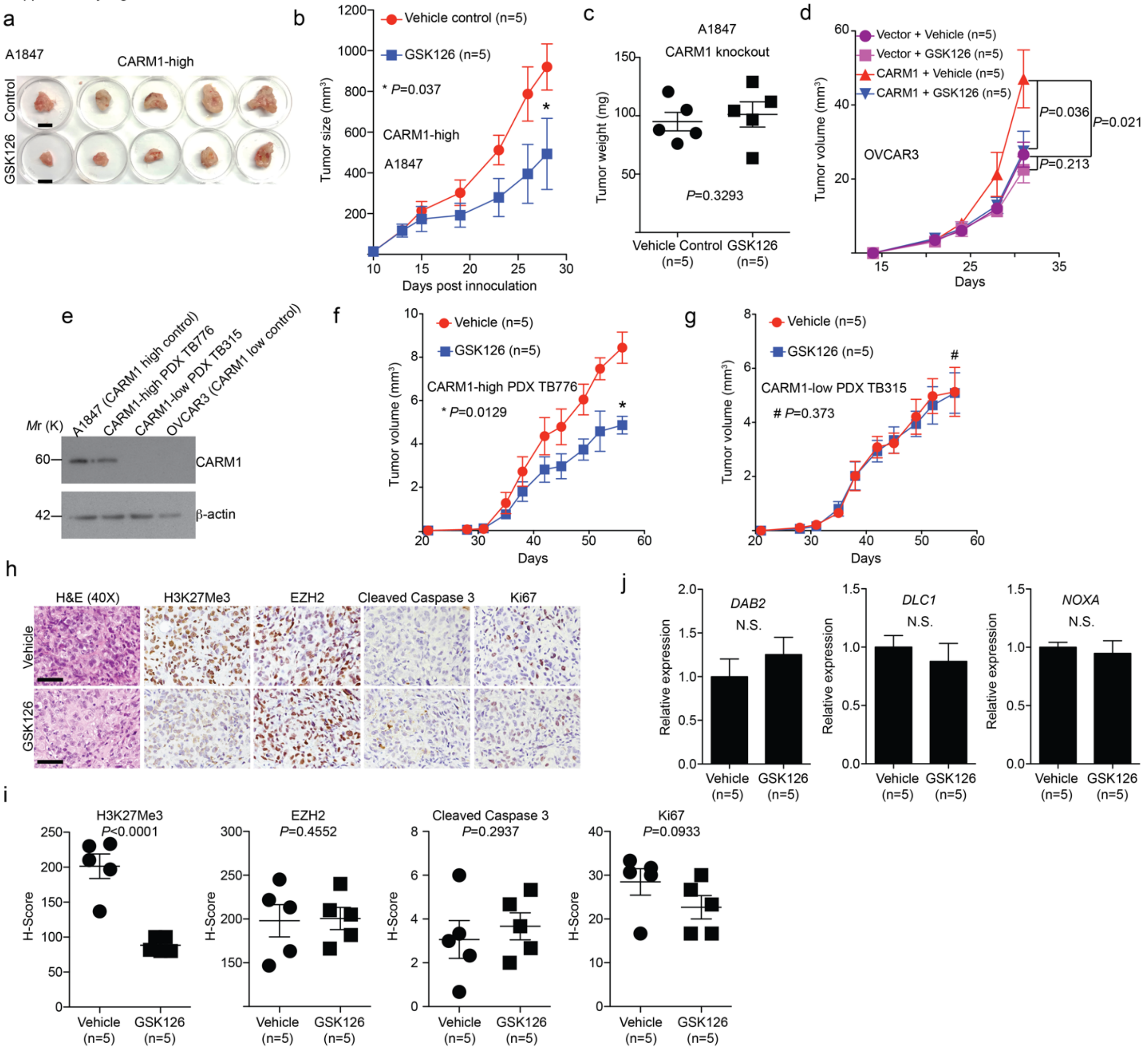

Supplementary Figure 6. EZH2 inhibition suppressed the growth of CARM1-expressing ovarian tumors *in vivo.*

(a) CARM1-high A1847 cells were injected subcutaneously into immunocompromised NSG mice (n=5/group). Tumors were allowed to establish for one week before the mice were randomized into two different treatment groups. Mice were treated with vehicle control or GSK126 (50 mg/kg daily) for an additional three weeks. At the end of treatment, the mice were euthanized. Shown are images of tumors dissected from control or GSK126 treated mice. Bar = 1cm.

(b) Same as a. Tumor size was measured as a surrogate for tumor burden from the control and GSK126 treated mice at the indicated time points.

(c) Same as b, but for CARM1 knockout A1847 cells. At the end of treatment, the mice were euthanized. Tumor weight was measured as a surrogate for tumor burden from the control and GSK126-treated mice.

(d) CARM1-low OVCAR3 cells with or without ectopic CARM1 expression were injected subcutaneously into immunocompromised NSG mice (n=5/group). Tumors were allowed to establish for one week before the mice were randomized into two different treatment groups. Mice were treated with vehicle control or GSK126 (50 mg/kg daily). Tumor size was measured as a surrogate for tumor burden from the control and GSK126 treated mice at the indicated time points.

(e-g) Expression of CARM1 and a loading control b-actin in the indicated CARM1-high and CARM1-low PDXs was determined by immunoblot (e). A1847 and OVCAR3 were used positive and negative controls, respectively. Mice with the indicated CARM1-high (f) and CARM-low (g) PDXs were randomized into two different treatment groups (n=5 mice/group) and treated with vehicle control or GSK126 (50 mg/kg daily). Tumor size was measured as a surrogate for tumor burden from the control and GSK126 treated mice at the indicated time points.

(h-j) Same as c. The serial sections of tumors dissected from the indicated treatment groups were subjected to immunohistochemical staining for H3K27Me3, EZH2, cleaved caspase 3 and Ki67 **(h)**. Scale bar = 50 μm. Histological score (H-score) of the indicated proteins was calculated for 3 separate fields from 5 tumors from 5 individual mice from each of the indicated groups **(i)**. Expression of the indicated EZH2/BAF155 target genes was determined by qRT-PCR in the tumors dissected from the indicated treatment groups **(j)**. Error bars represent SEM. N.S.: not significant. *P*-values are from two-tailed *t*-test.

Figure 1

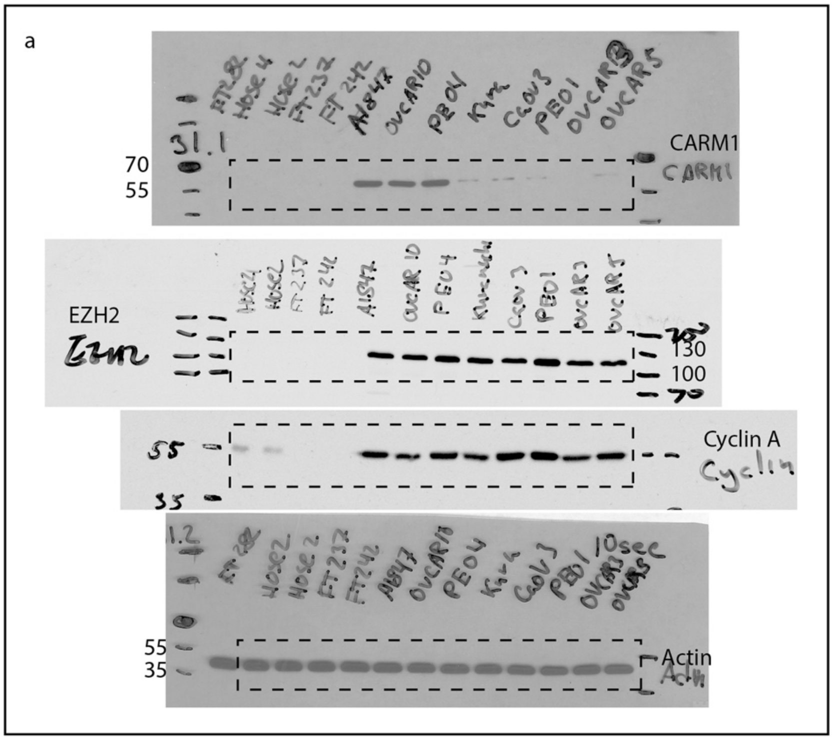

Figure 1

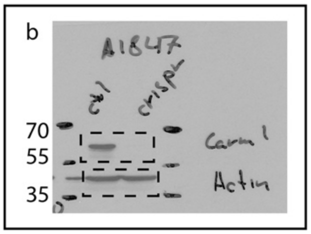

Figure 1

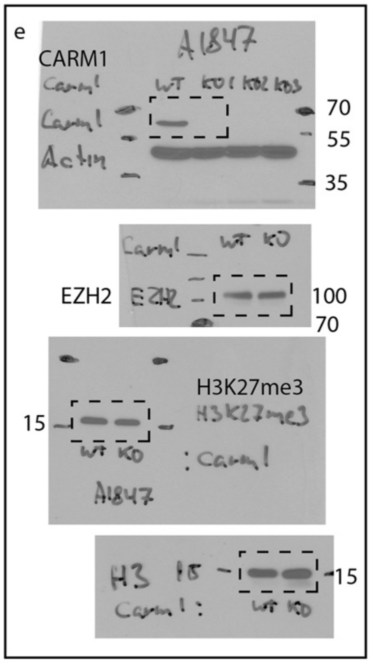

Figure 2

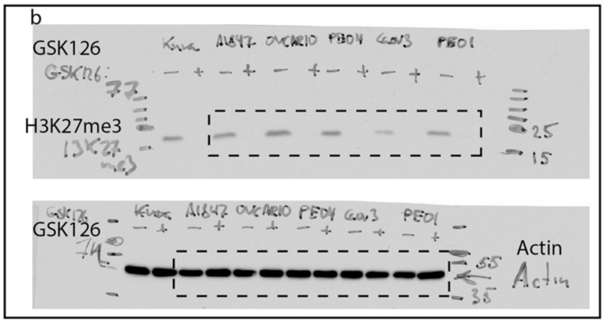

Figure 2

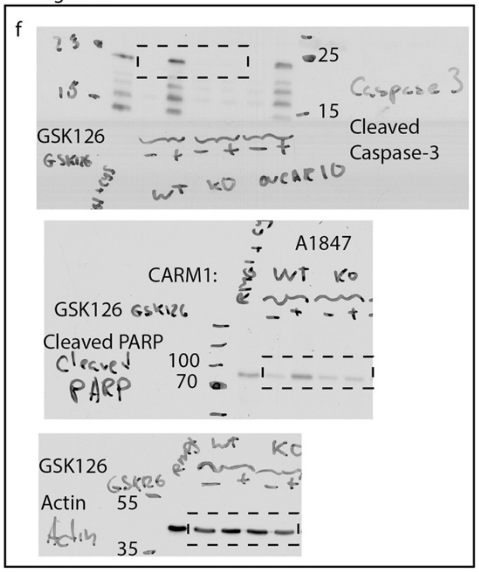

Figure 2

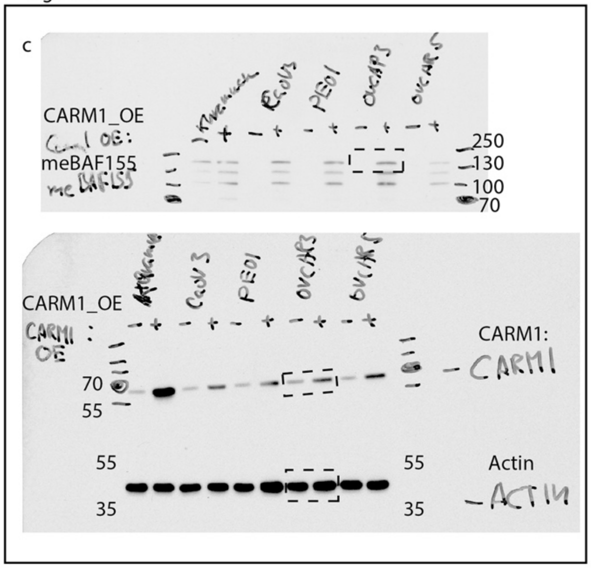

Figure 2

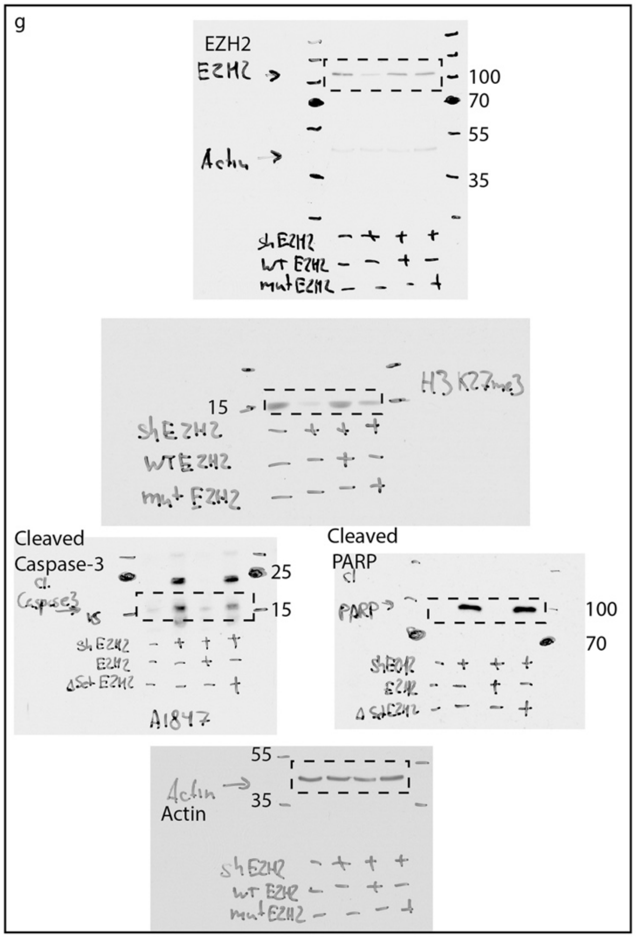

Figure 3

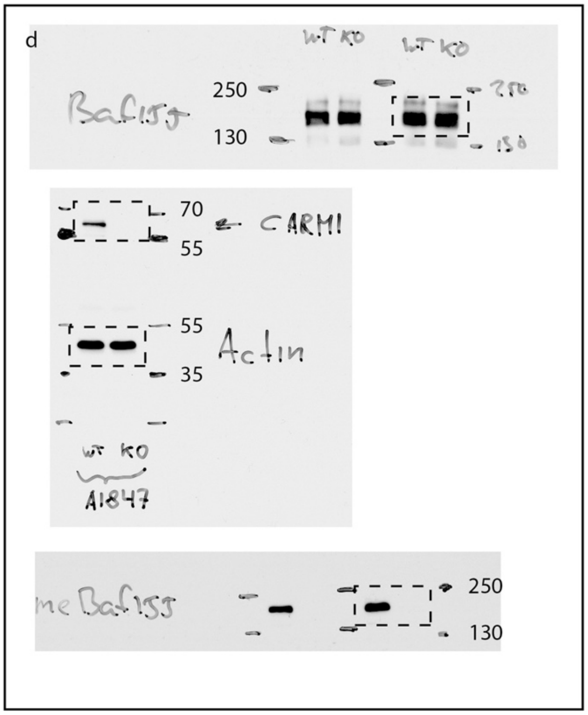

Figure 5

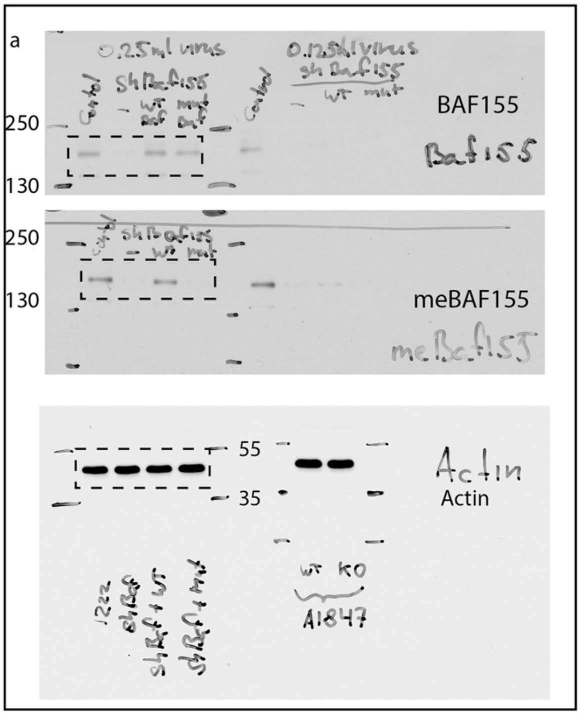

Supplemental Figure 1

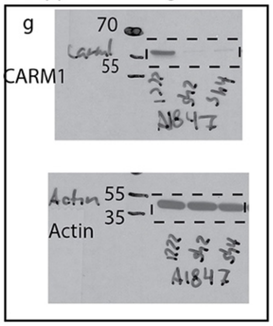

Supplemental Figure 2

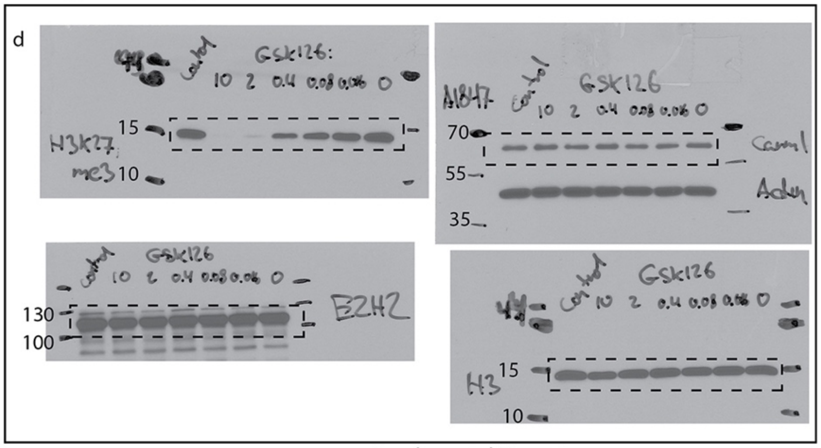

Supplemental Figure 1

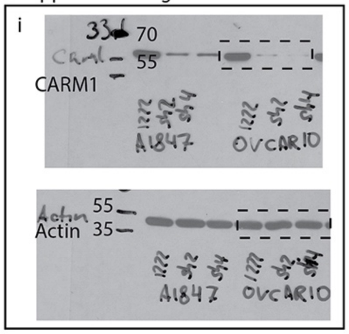

Supplemental Figure 2

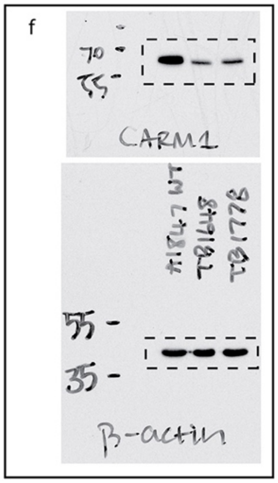

Supplemental Figure 2

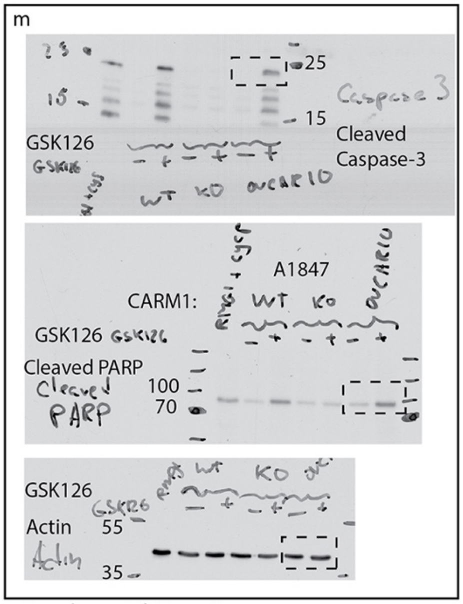

Supplemental Figure 2

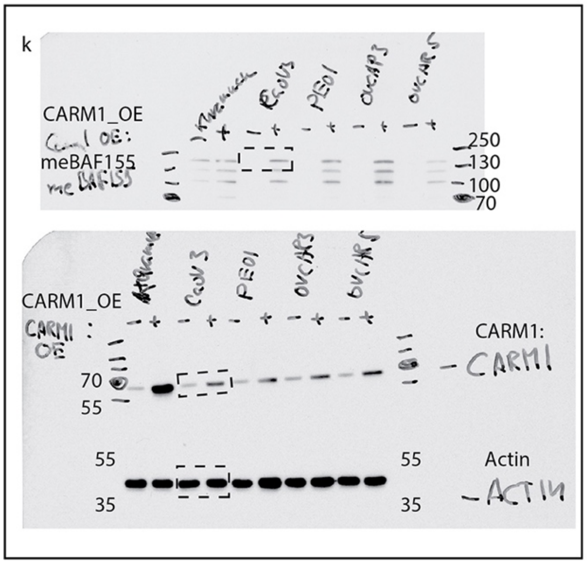

Supplemental Figure 2

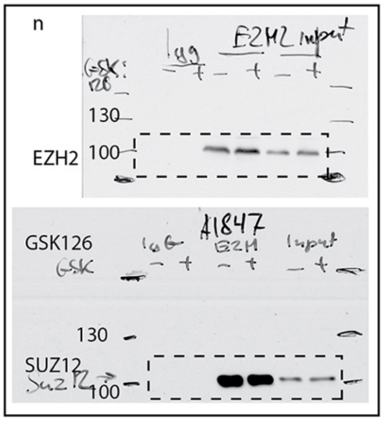

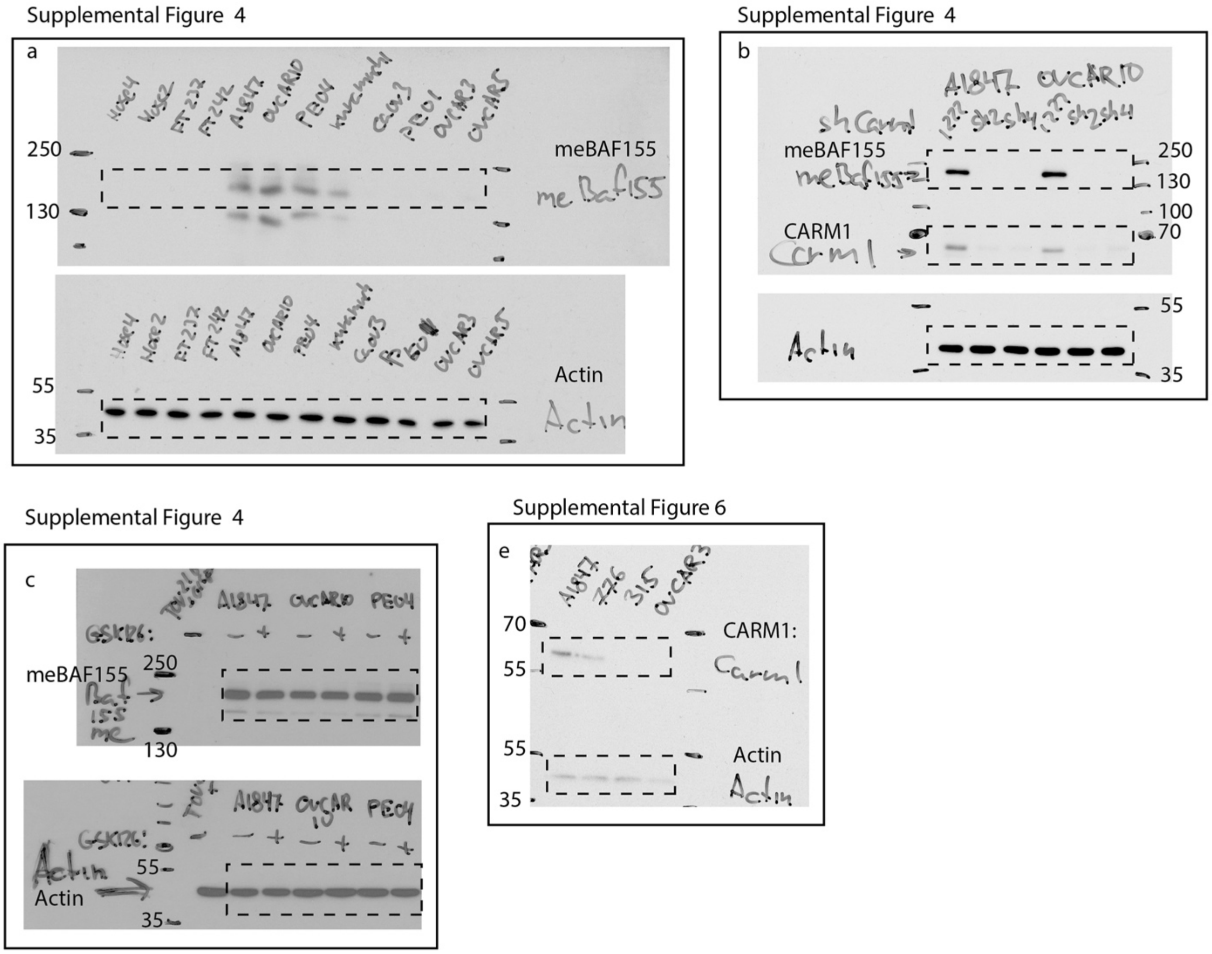

Supplementary Figure 7. Unprocessed images of blots. Unprocessed images of scanned immunoblots shown in Figures and Supplementary Figures are provided.

Supplementary Table 1. EZH2 inhibitors are selective against CARM1 expression							
			CARM1 High Parental A1847		CARM1 Knockout		
Compound	Target	Dose, µM	Relative Growth	S.E.M.	Relative Growth	S.E.M.	*P*-value
A366	G9a, GLP	20	1.12610177	0.026994901	1.083216187	0.024462549	0.284100154
Bromosporine	pan-Bromodomain	20	0.00076137	0.00076137	0.013530824	0.011493706	0.347865117
C1994	CREBBP/EP300	3.6	0.00010305	0.000103051	0.031107943	0.014268088	0.118116966
C646	CREBBP, EP300	0.8	0.92105820	0.01489038	1.102442703	0.013846102	0.000113966
CBP112	CREBBP, EP300	7.8	0.94978798	0.026387138	0.65895342	0.209455497	0.259485988
CBP30	HDAC1	20	0.99663221	0.018982712	0.54380163	0.040955842	0.000416278
GSK/LSD1	LSD1	20	0.98728639	0.006584402	1.007863957	0.034432096	0.595855393
DMSO	Vehicle Control	0	0.95170977	0.023908506	1.062526654	0.030591842	0.030917171
**GSK126**	**EZH2**	**10**	**0.15651653**	**0.080547528**	**0.532314948**	**0.051413606**	**0.010631224**
GSK2901	BAZ2A, BAZ2B	0.4	0.89260249	0.018578154	1.170477089	0.021930676	8.23709E-05
GSKJ4	JMJD3, UTX	0.61	1.16061609	0.018984972	1.063614933	0.008768788	0.008546659
IOX1	pan-2-OG	16	0.03557553	0.010172418	0.281729478	0.034844998	0.003916368
IOX2	PHD2	0.03	1.05962417	0.028477162	1.020770482	0.035433688	0.426973425
JQ1	BRD2, BRD3, BRD4, BRDT (BET)	20	0.00115055	0.000340774	0.007319818	0.004529224	0.26655804
LAQ824	HDACs	0.02	0.00009757	9.75693E-05	0.004824201	0.001825039	0.080873167
LLY-507	SMYD2	2.7	1.04802932	0.029072456	0.856154144	0.023618675	0.002454216
Olaparib	PARP	0.7	0.01828986	0.014278756	0.02455379	0.008317064	0.720739487
PFI-1	BRD2, BRD3, BRD4, BRDT (BET)	0.8	0.19467600	0.021110016	0.013219267	0.012196005	0.000821766
PFI-2	SETD7	20	0.95709362	0.028031604	1.0820438	0.045106733	0.065165254
PFI-3	SMARCA,PB1	20	0.90866744	0.030985436	1.104246892	0.011560561	0.004730005
UNC0638	G9a, GLP	20	0.08062460	0.011946064	0.047423828	0.016990494	0.166662741
UNC0642	G9a, GLP	8	0.24296953	0.039794339	0.015929696	0.009532955	0.008612528
UNC1215	HDAC6, HDAC2, HDAC3, HDAC8	20	0.92996441	0.039479602	1.046045711	0.031762606	0.063852099
**UNC1999**	**EZH2**	**0.48**	**0.56224648**	**0.058501852**	**0.761331101**	**0.037563764**	**0.034353525**

Supplementary Table 1. EZH2 inhibitors are selective against CARM1 expression. CARM1-high parental controls and CARM1 knockout A1847 cells were treated with the indicated concentration of the small molecules in a 14-days colony formation assay (list alphabetically). Integrated density for each well was calculated using NIH Image J software. The relative growth of the indicated cells was determined by normalizing to the vehicle control treated cells. n=4 and *P*-values are from two-tailed *t*-test.

Supplementary Table 2. Biologically relevant EZH2 target genes that are downregulated by CARM1																
		Correlation between with CARM1 expression in TCGA samples				mRNA expression							ChIP-seq signal intensity			
		Pearson correlation		Spearman correlation		Control/CARM1 knockout			RNA seq count		RNA seq FPKM		Control		CARM1 knockout	
#	Gene	R value	p-value	R value	p-value	fold change	p-value	fdr	Control	CARM1 KO	Control	CARM1 KO	EZH2	H3K27me3	EZH2	H3K27me3
1	MAP3K5	-0.198965049	4.08177E-06	-0.217951734	4.25453E-07	-3.185196512	5.45E-09	0%	881	4428	4.29	18.12	4.71	4.90	0.00	0.00
2	PMAIP1	-0.175084537	5.23484E-05	-0.175419055	5.06219E-05	-3.382108093	3.6E-10	0%	153	544	1.14	3.88	3.28	4.68	0.00	0.00
3	TGFBI	-0.156839732	0.000297147	-0.14902825	0.000591471	-4.296812722	1.3E-16	0%	110	465	0.75	3.04	3.01	4.41	0.00	0.00
4	DAB2	-0.13373293	0.002073418	-0.147861187	0.000653711	-3.92934096	1.3E-11	0%	106	438	0.99	4.79	4.05	4.61	0.00	0.00
5	ITGA4	-0.139887941	0.001270235	-0.142587487	0.001018204	-3.184665486	5.8E-10	0%	239	800	3.05	8.65	3.71	3.15	0.00	0.00
6	RND3	-0.167642586	0.000108644	-0.140071566	0.00125142	-5.93743752	1.4E-22	0%	6	50	0.17	1.7	3.33	5.90	0.00	0.00
7	PPARGC1A	-0.116077901	0.007585654	-0.139752622	0.001284266	-23.86194957	1.5E-06	0%	1	28	0.01	0.42	3.50	3.49	0.00	0.00
8	DLC1	-0.140648915	0.001193924	-0.133147796	0.002170058	-3.555559726	7.3E-13	0%	1027	3837	6.56	20.43	5.10	5.94	0.00	0.00
9	PDE4B	-0.092141219	0.034282702	-0.130370969	0.002687269	-14.31136541	3E-16	0%	8	122	0.16	2.37	5.40	5.45	0.00	0.00
10	FMN2	-0.12600036	0.003731964	-0.121151589	0.005311183	-4.572896825	2.7E-14	0%	115	553	2.22	9.63	3.26	3.69	0.00	0.00
11	MME	-0.104759346	0.016035316	-0.111333348	0.010462821	-4.366394913	2.4E-15	0%	280	1285	2.76	11.66	5.14	4.79	0.00	0.00
12	TNC	-0.110565685	0.011009917	-0.106999047	0.013897985	-12.75997307	2.2E-29	0%	619	3001	3.94	17.78	4.53	3.37	0.00	-3.60
13	KLF5	-0.11041769	0.011118258	-0.104742287	0.016052644	-4.054372234	1E-14	0%	541	2305	8.15	37.42	3.20	5.51	0.00	0.00
14	EPHA4	-0.086673905	0.046520259	-0.100322606	0.021133339	-8.265600453	2.3E-20	0%	30	261	0.33	2.76	3.59	6.07	0.00	0.00
15	ANTXR1	-0.094541709	0.02984649	-0.099743744	0.021892744	-4.641694733	1.2E-16	0%	299	946	1.8	5.43	3.91	3.20	0.00	0.00
16	FN1	-0.116142316	0.007552021	-0.099172431	0.022665348	-14.58093837	2E-45	0%	1	19	0.04	0.71	4.65	3.78	0.00	0.00
17	ANTXR2	-0.091802828	0.034951143	-0.095685501	0.027911995	-3.071935896	4.6E-10	0%	6	38	0.15	0.88	5.87	4.01	0.00	0.00
18	GPR37	-0.098490018	0.023619066	-0.091042741	0.036493133	-3.051285042	0.00013	0%	23	74	0.26	0.81	4.40	4.40	0.00	0.00
19	TGFBR2	-0.087770064	0.043808917	-0.087845362	0.043627654	-3.313985283	9.3E-11	0%	294	1024	2.15	7.14	5.76	3.99	0.00	0.00

Supplementary Table 2. The list of the 19 identified EZH2/H3K27Me3 target apoptosis-promoting genes that are upregulated by CARM1 knockout and negatively correlates with CARM1 expression in the 579 TCGA high grade serous ovarian carcinoma database.

Supplementary Table 3.
Top 10 upstream transcription factors that are enriched by the differentially expressed genes.

Upstream transcription Regulator	p-value of overlap	Number of genes
SMARCA4	1.22E-30	175
CTNNB1	7.00E-28	190
JUN	2.71E-18	119
CREB1	5.16E-18	140
TP53	2.94E-17	280
SOX2	6.43E-17	94
NFKBIA	5.84E-16	115
STAT3	9.43E-16	130
NEUROG1	9.79E-16	35
NKX2-3	1.44E-15	71

Supplementary Table 3. The top 10 upstream transcription factors that are enriched by the differentially expressed gene in control parental and CARM1 knockout A1847 cells determined by Ingenuity pathway analysis.

References Cited

1. Cancer Genome Atlas Research, N. Integrated genomic analyses of ovarian carcinoma. *Nature* **474**, 609-615 (2011).
2. Mok, S.C.*, et al.* A gene signature predictive for outcome in advanced ovarian cancer identifies a survival factor: microfibril-associated glycoprotein 2. *Cancer Cell* **16**, 521-532 (2009).
3. Tone, A.A.*, et al.* Gene expression profiles of luteal phase fallopian tube epithelium from BRCA mutation carriers resemble high-grade serous carcinoma. *Clin Cancer Res* **14**, 4067-4078 (2008).
4. Bild, A.H.*, et al.* Oncogenic pathway signatures in human cancers as a guide to targeted therapies. *Nature* **439**, 353-357 (2006).
5. Abel, U., Berger, J. & Wiebelt, H. CRITLEVEL: an exploratory procedure for the evaluation of quantitative prognostic factors. *Methods Inf Med* **23**, 154-156 (1984).
6. Zhou, X., Thorgeirsson, S.S. & Popescu, N.C. Restoration of DLC-1 gene expression induces apoptosis and inhibits both cell growth and tumorigenicity in human hepatocellular carcinoma cells. *Oncogene* **23**, 1308-1313 (2004).
